# Supplementary figures and images for: Deletion of the scavenger receptor Scarb1 in osteoblast progenitors and myeloid cells does not affect bone mass
Source: PLoS One. 2025 Oct 31;20(10):e0328754. doi: 10.1371/journal.pone.0328754 (PMC12578142; doi:10.1371/journal.pone.0328754)

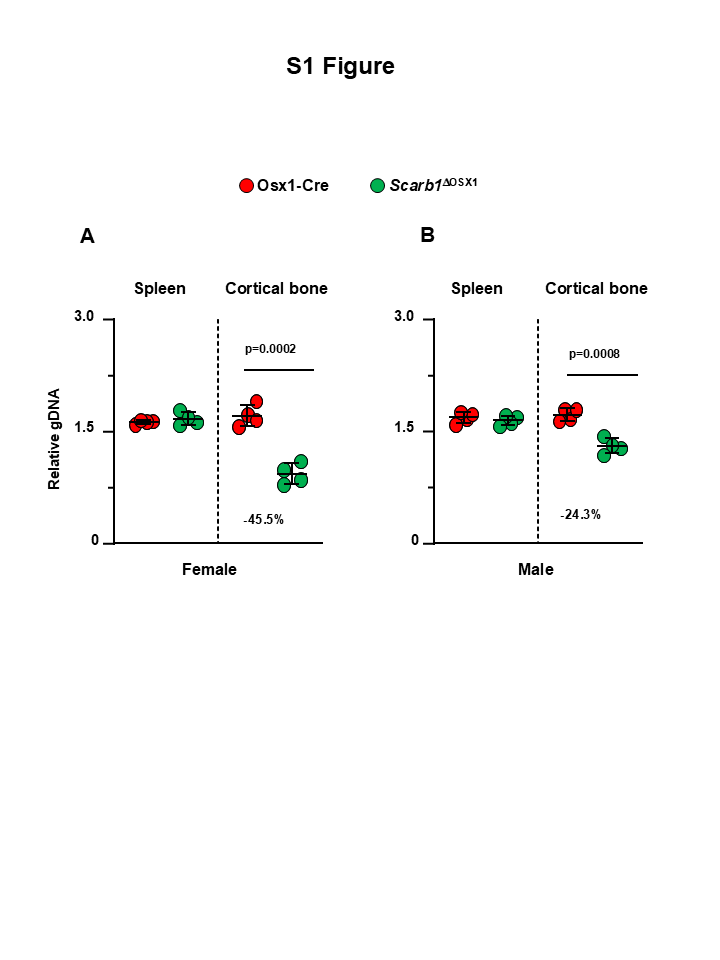

Supplement: S1 Fig — Quantitative PCR (qPCR) of genomic DNA isolated from femoral and tibial cortical bone and spleen in 6-month-old females [Osx1-Cre n = 4, Osx1-Cre; Scarb1ΔOSX1n=4] (A) and in 6-month-old males [Osx1-Cre n = 4, Scarb1ΔOSX1n=4] (B). Data are shown as mean and standard deviation. Data analyzed by unpaired T-test. (TIF) [file pone.0328754.s001.TIF]

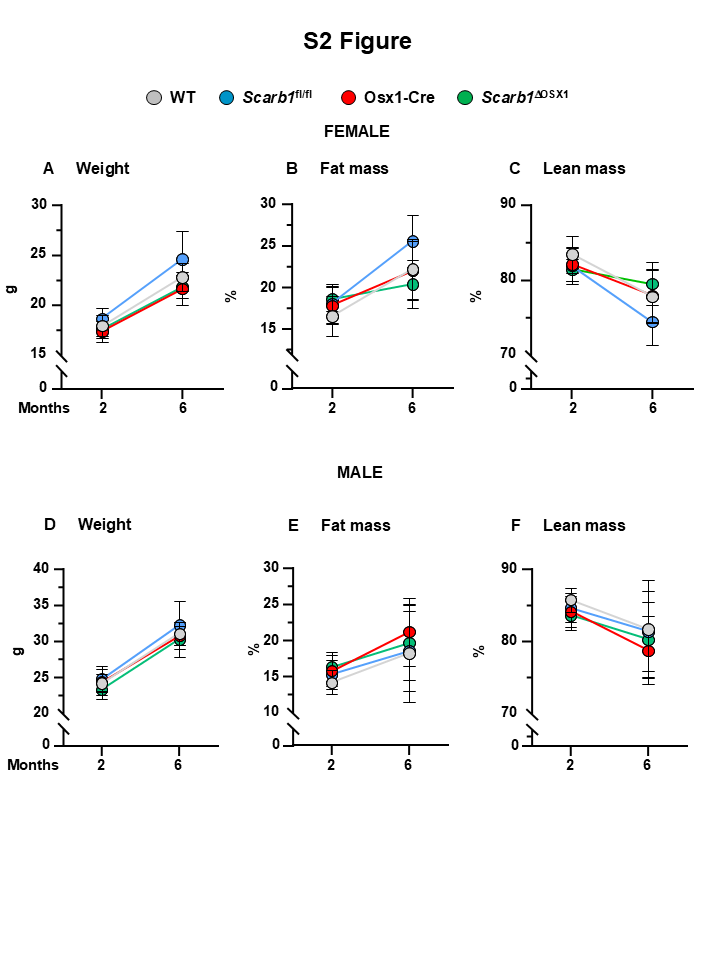

Supplement: S2 Fig — Measurements of weight, fat mass and lean mass in 2- and 6-month-old females [WT n = 17; Scarb1fl/fl n = 9; Osx1-Cre n = 14; Scarb1ΔOSX1n=10] (A-C) and 2- and 6-month-old males [WT n = 11; Scarb1fl/fl n = 14; Osx1-Cre n = 12; Scarb1ΔOSX1n=12] (D-F). Data are shown as mean and standard deviation. Adjusted p-values <0.05, calculated by repeated measures using two-way ANOVA, are shown in S3 Table. (TIF) [file pone.0328754.s002.TIF]

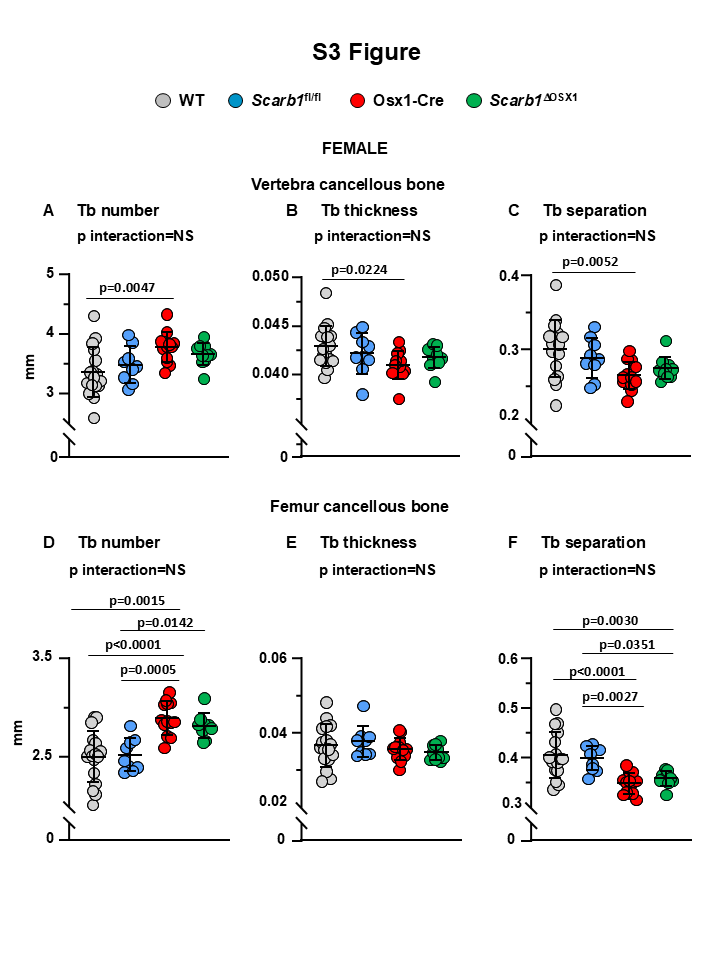

Supplement: S3 Fig — Micro-CT analysis of cancellous bone architecture in 6-month-old females. (A) Trabecular number, (B) trabecular thickness, and (C) trabecular separation of vertebral cancellous bone. (D) Trabecular number, (E) trabecular thickness, and (F) trabecular separation of femoral cancellous bone [WT n = 17; Scarb1fl/fl n = 9; Osx1-Cre n = 13; Scarb1ΔOSX1n=10]. Data are shown as mean and standard deviation. Data analyzed by 2-way ANOVA; the p-values were adjusted using the Tukey’s pairwise comparison procedure. Tb, trabecular. (TIF) [file pone.0328754.s003.tif]

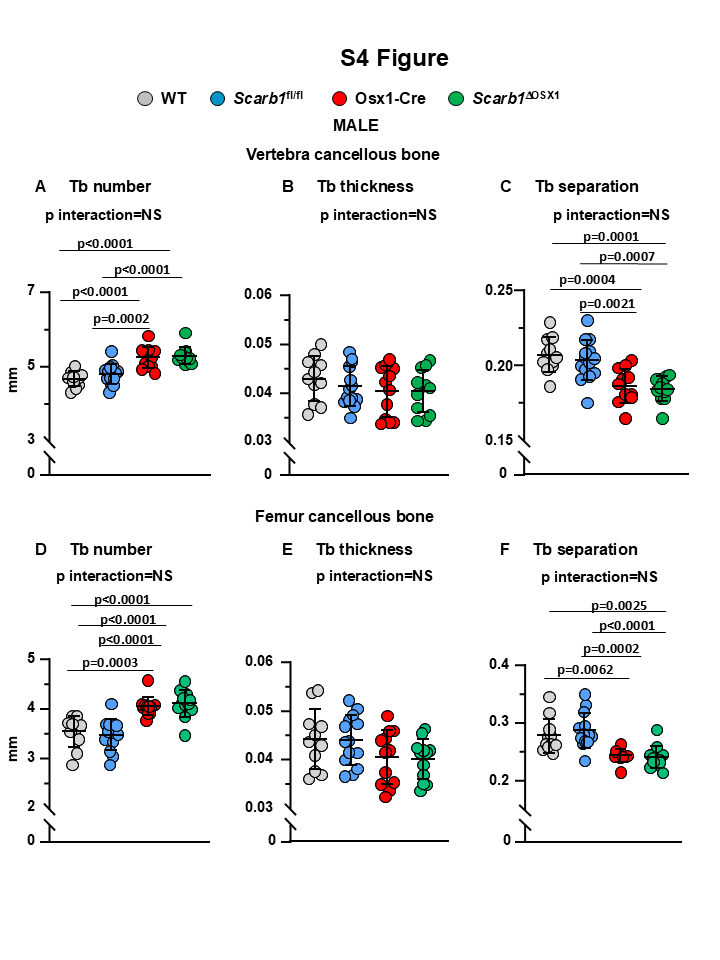

Supplement: S4 Fig — Micro-CT analysis of cancellous bone architecture in 6-month-old males. (A) Trabecular number, (B) trabecular thickness, and (C) trabecular separation of vertebral cancellous bone. (D) Trabecular number, (E) trabecular thickness, and (F) trabecular separation of femoral cancellous bone [WT n = 11; Scarb1fl/fl n = 14; Osx1-Cre n = 12; Scarb1ΔOSX1n=12]. Data are shown as mean and standard deviation. Data analyzed by 2-way ANOVA; the p-values were adjusted using the Tukey’s pairwise comparison procedure. Tb, trabecular. (TIF) [file pone.0328754.s004.TIF]

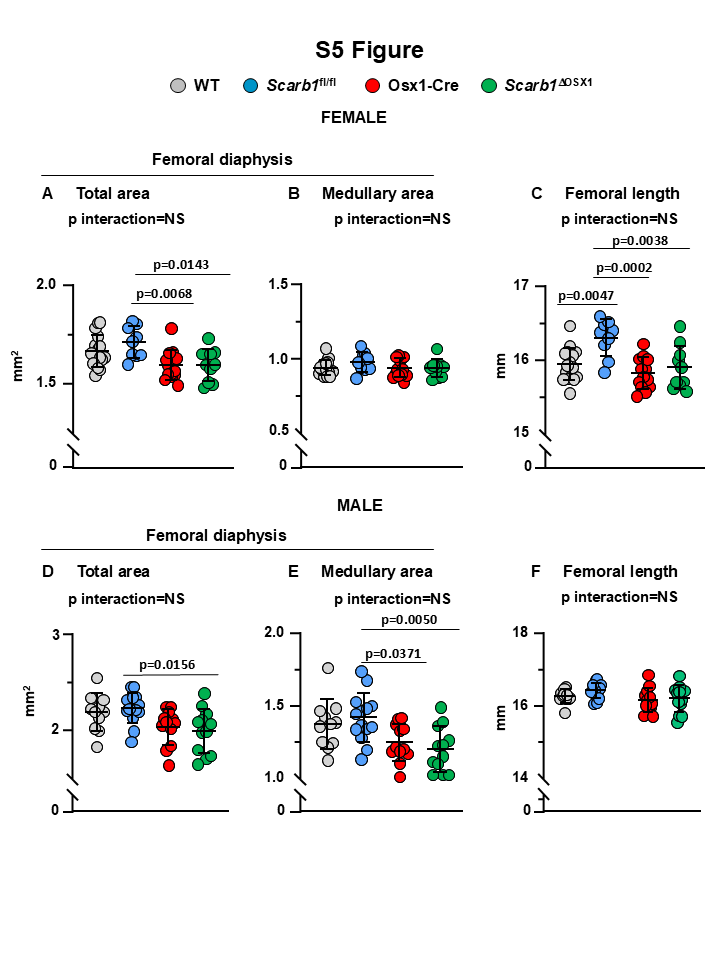

Supplement: S5 Fig — Micro-CT analysis of cortical bone architecture and femoral length. (A) Total area, (B) Medullary area, (C) Femoral length in 6-month-old females [WT n = 17; Scarb1fl/fl n = 9; Osx1-Cre n = 13; Scarb1ΔOSX1n=10]. (D) Total area, (E) Medullary area, (F) Femoral length in 6-month-old males [total area and medullary area WT n = 11; Scarb1fl/fl n = 14; Osx1-Cre n = 12; Scarb1ΔOSX1n=12], [femoral length WT n = 11; Scarb1fl/fl n = 13, Osx1-Cre n = 12; Scarb1ΔOSX1n=12]. Data are shown as mean and standard deviation. Data analyzed by 2-way ANOVA; the p-values were adjusted using the Tukey’s pairwise comparison procedure. (TIF) [file pone.0328754.s005.TIF]

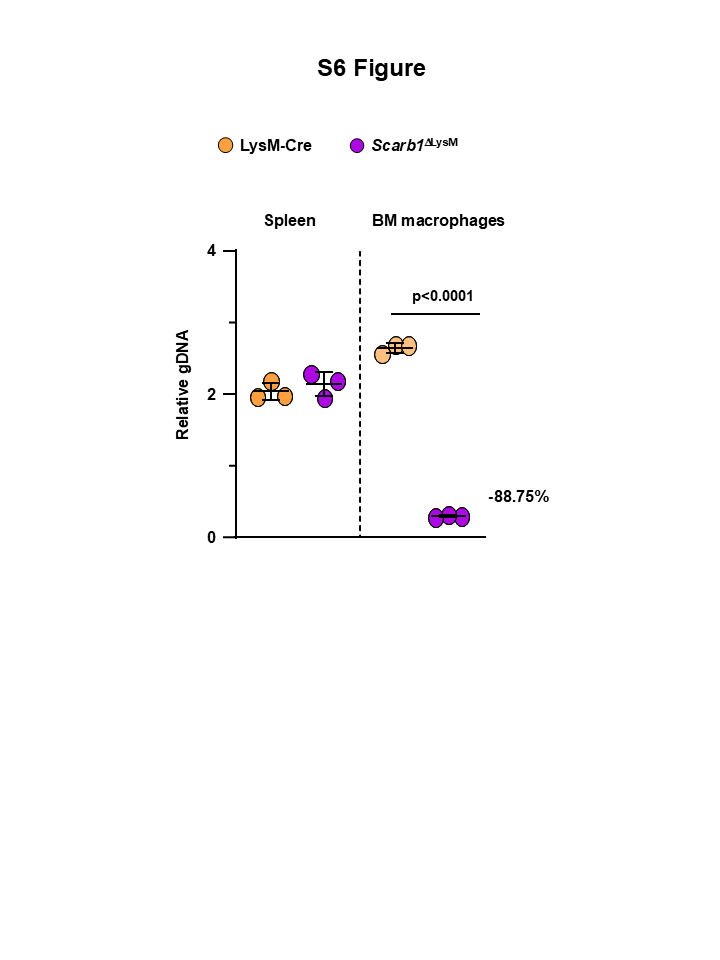

Supplement: S6 Fig — Quantitative PCR (qPCR) of genomic DNA isolated from bone marrow-derived macrophages and spleen of 6-month-old female mice [LysM-Cre n = 3, Scarb1ΔLysM n = 3]. Data are shown as mean and standard deviation. Data analyzed by unpaired T-test. (TIF) [file pone.0328754.s006.TIF]

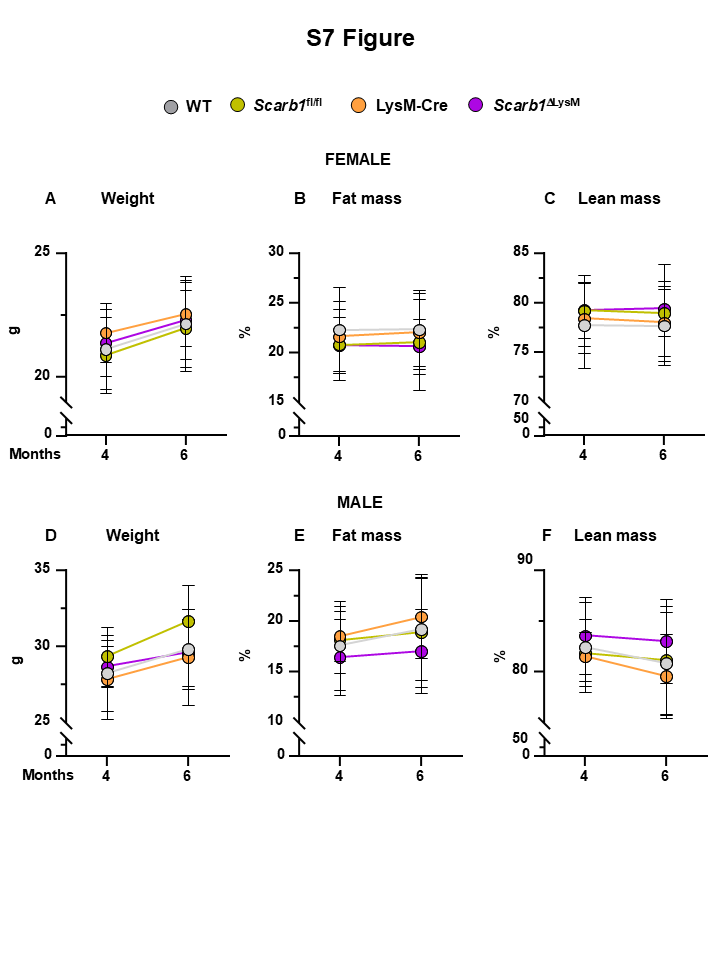

Supplement: S7 Fig — Measurements of weight, fat mass and lean mass in 4- and 6-month-old females [WT n = 12; Scarb1fl/fl n = 8; LysM-Cre n = 15; Scarb1ΔLysM n = 12] (A-C) and 4- and 6-month-old males [WT n = 14; Scarb1fl/fl n = 14; LysM-Cre n = 11; Scarb1ΔLysM n = 16]. Data are shown as mean and standard deviation. Adjusted p-values <0.05, calculated by repeated measures using two-way ANOVA, are shown in S3 Table. (TIF) [file pone.0328754.s007.TIF]

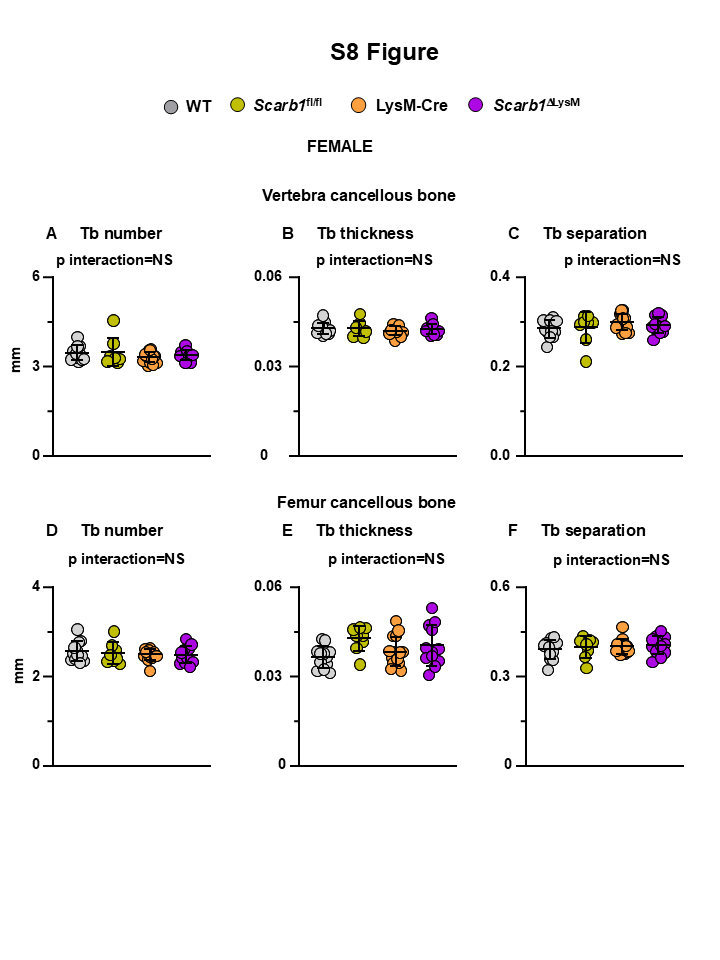

Supplement: S8 Fig — Micro-CT analysis of cancellous bone architecture in 6-month-old females. (A) Trabecular number, (B) trabecular thickness, and (C) trabecular separation of vertebral cancellous bone. (D) Trabecular number, (E) trabecular thickness, and (F) trabecular separation of femoral cancellous bone [WT n = 12; Scarb1fl/fl n = 8; LysM-Cre n = 15; Scarb1ΔLysM n = 12]. Data are shown as mean and standard deviation. Data analyzed by 2-way ANOVA; the p-values were adjusted using the Tukey’s pairwise comparison procedure. Tb, trabecular. (TIF) [file pone.0328754.s008.TIF]

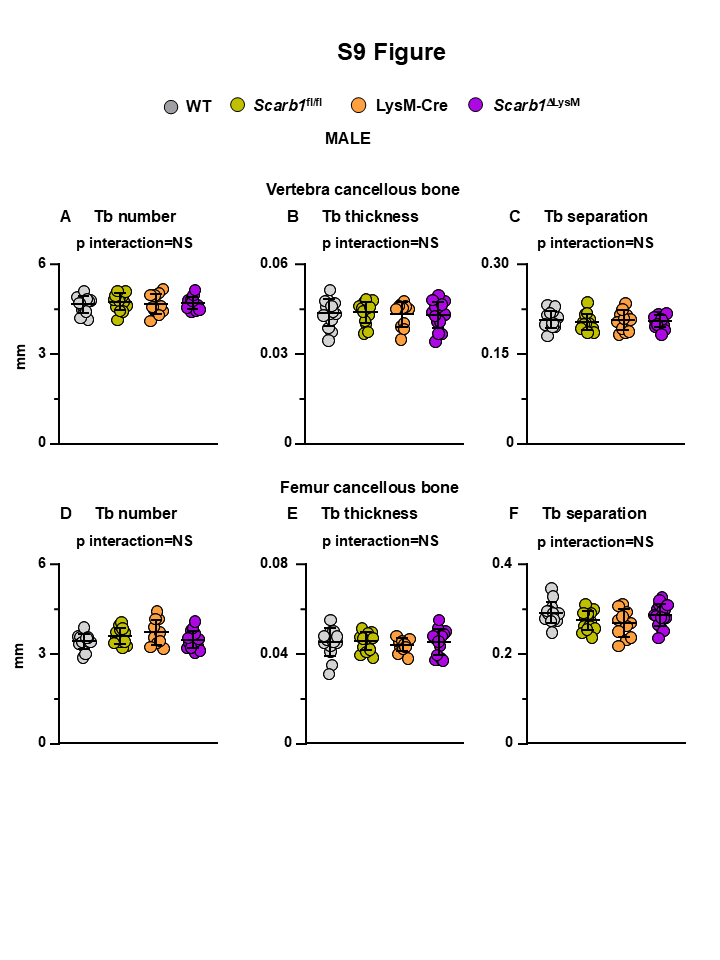

Supplement: S9 Fig — Micro-CT analysis of cancellous bone architecture in 6-month-old males. (A) Trabecular number, (B) trabecular thickness, and (C) trabecular separation of vertebral cancellous bone. (D) Trabecular number, (E) trabecular thickness, and (F) trabecular separation of femoral cancellous bone [WT n = 14; Scarb1fl/fl n = 14; LysM-Cre n = 11; Scarb1ΔLysM n = 16]. Data are shown as mean and standard deviation. Data analyzed by 2-way ANOVA; the p-values were adjusted using the Tukey’s pairwise comparison procedure. Tb, trabecular. (TIF) [file pone.0328754.s009.TIF]

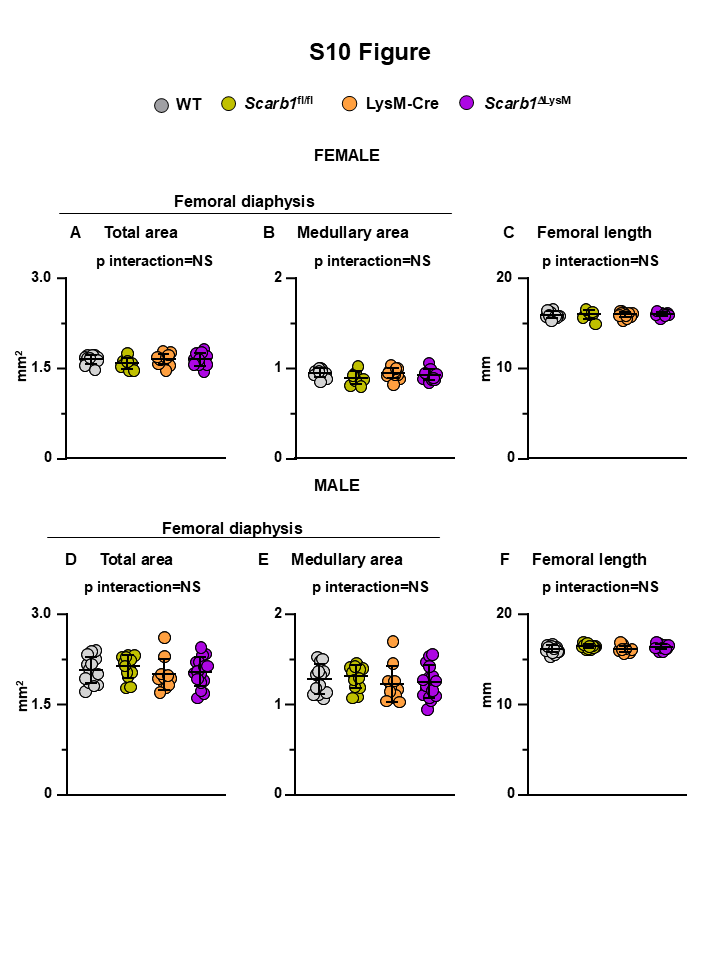

Supplement: S10 Fig — Micro-CT analysis of cortical bone architecture and femoral length. (A) Total area, (B) Medullary area, (C) Femoral length in 6-month-old females [WT n = 12; Scarb1fl/fl n = 8; LysM-Cre n = 15; LysM-Cre; Scarb1fl/fl n = 12], (D) Total area, (E) Medullary area, (F) Femoral length in 6-month-old males [WT n = 14; Scarb1fl/fl n = 14; LysM-Cre n = 11; Scarb1ΔLysM n = 16]. Data are shown as mean and standard deviation. Data analyzed by 2-way ANOVA; the p-values were adjusted using the Tukey’s pairwise comparison procedure. (TIF) [file pone.0328754.s010.TIF]
