# Supplementary material for: Deletion of the scavenger receptor Scarb1 in osteoblast progenitors and myeloid cells does not affect bone mass
Source: PLoS One. 2025 Oct 31;20(10):e0328754. doi: 10.1371/journal.pone.0328754 (PMC12578142; doi:10.1371/journal.pone.0328754)
Supplement: S1 Table — List of the TaqMan primers used for quantification of mRNA and genomic DNA by qPCR. (DOCX) [file pone.0328754.s011.docx]

**S1 Table**

| **NCBI gene name** | **Gene name** | **TaqMan #** |
| --- | --- | --- |
| ***Bglap*** | bone gamma-carboxyglutamate protein | Mm03413826_mH |
| ***Alpl*** | alkaline phosphatase 2 | Mm00475831_m1 |
| ***Mrps2*** | mitochondrial ribosomal protein S2 | Mm00475529_m1 |
| ***Tfrc*** | transferrin receptor gene | 4458366 |
| ***Scarb1* Exon 2** | scavenger receptor class B, member 1 | Fwd 5’- GGACTGTGTGTGGGTGTGT-3’  Rev: 5’- TTCTGTCTCTGGAGCAATCAATCTC-3’  Probe: 5’- CTGCCATGCTGAGTTTT-3’ |
