# Supplementary material for: Deletion of the scavenger receptor Scarb1 in osteoblast progenitors and myeloid cells does not affect bone mass
Source: PLoS One. 2025 Oct 31;20(10):e0328754. doi: 10.1371/journal.pone.0328754 (PMC12578142; doi:10.1371/journal.pone.0328754)
Supplement: S2 Table — (DOCX) [file pone.0328754.s012.docx]

|  | **comparison** | **p- value** |
| --- | --- | --- |
| **Fig 2A Spine BMD** | p- interaction (*Scarb1*^fl/fl^ vs Osx1-Cre vs time) | 0.005 |
| **Fig 2B Femoral BMD** | Osx1-Cre and *Scarb1*^ΔOSX1^ vs WT and *Scarb1*^fl/fl^ | 0.0007 |
| **Fig 2C Total BMD** | Osx1-Cre and *Scarb1*^ΔOSX1^ vs WT and *Scarb1*^fl/fl^ | 0.0012 |
|  | *Scarb1*^fl/fl^ and *Scarb1*^ΔOSX1^ vs WT and Osx1-Cre vs time | 0.0007 |
| **Fig 3B Femoral BMD** | Osx1-Cre and *Scarb1*^ΔOSX1^ vs WT and *Scarb1*^fl/fl^ | 0.043 |
|  | *Scarb1*^fl/fl^ and *Scarb1*^ΔOSX1^ vs WT and Osx1-Cre vs time | 0.046 |
| **Fig 3C Total BMD** | Osx1-Cre and *Scarb1* ^ΔOSX1^ vs WT and *Scarb1*^fl/fl^ | 0.037 |
| **Fig 4A Spine BMD** | *Scarb1*^fl/fl^ and *Scarb1*^ΔLysM^ vs WT and LysM-Cre vs time | 0.036 |
| **Fig 4C Total BMD** | p- interaction (*Scarb1*^fl/fl^ vs LysM-Cre vs time) | 0.014 |

**S2 Table**
