# Supplementary material for: Deletion of the scavenger receptor Scarb1 in osteoblast progenitors and myeloid cells does not affect bone mass
Source: PLoS One. 2025 Oct 31;20(10):e0328754. doi: 10.1371/journal.pone.0328754 (PMC12578142; doi:10.1371/journal.pone.0328754)
Supplement: S3 Table — (DOCX) [file pone.0328754.s013.docx]

**S3 Table**

|  | **comparison** | **p- value** |
| --- | --- | --- |
| **S2 Fig panel A weight** | Osx1-Cre and *Scarb1*^ΔOSX1^ vs WT and *Scarb1*^fl/fl^ | 0.0005 |
|  | Osx1-Cre and *Scarb1*^ΔOSX1^ vs WT and *Scarb1*^fl/fl^ vs time | 0.004 |
|  | *Scarb1*^fl/fl^ and *Scarb1*^ΔOSX1^ vs WT and Osx1-Cre | 0.047 |
| **S2 Fig panel B fat mass** | Osx1-Cre and *Scarb1*^ΔOSX1^ vs WT and *Scarb1*^fl/fl^ | 0.030 |
| **S2 Fig panel C lean mass** | Osx1-Cre and *Scarb1*^ΔOSX1^ vs WT and *Scarb1*^fl/fl^ | 0.030 |
| **S2 Fig panel E fat mass** | Osx1-Cre and *Scarb1*^ΔOSX1^ vs WT and *Scarb1*^fl/fl^ | 0.004 |
| **S2 Fig panel F lean mass** | Osx1-Cre and *Scarb1*^ΔOSX1^ vs WT and *Scarb1*^fl/fl^ | 0.004 |
| **S7 Fig panel D weight** | p- interaction (*Scarb1*^fl/fl^ vs LysM-Cre vs time) | 0.033 |
|  | LysM-Cre and *Scarb1*^ΔLysM^ vs WT and *Scarb1*^fl/fl^ | 0.021 |
|  | LysM-Cre and *Scarb1*^ΔLysM^ vs WT and *Scarb1*^fl/fl^ vs time | 0.003 |
|  | *Scarb1*^fl/fl^ and *Scarb1*^ΔLysM^ vs WT and LysM-Cre | 0.015 |
