# Supplementary material for: Deletion of the scavenger receptor Scarb1 in osteoblast progenitors and myeloid cells does not affect bone mass
Source: PLoS One. 2025 Oct 31;20(10):e0328754. doi: 10.1371/journal.pone.0328754 (PMC12578142; doi:10.1371/journal.pone.0328754)
Supplement: Attachment — Submitted filename: RESPONSE TO REVIEWERS.docx [file pone.0328754.s015.docx]

Dear Dr Roberts,

We appreciate the editor and reviewer’s constructive criticism and the opportunity to revise our manuscript. In the revised version, we have addressed all the concerns raised to the best of our ability. Our responses are shown below in blue text.

Response to the Editorial requirements:

1. Please ensure that your manuscript meets PLOS ONE's style requirements, including those for file naming. The PLOS ONE style templates can be found at https://journals.plos.org/plosone/s/file?id=wjVg/PLOSOne_formatting_sample_main_body.pdf and https://journals.plos.org/plosone/s/file?id=ba62/PLOSOne_formatting_sample_title_authors_affiliations.pdf

Those requirements have been checked and all the documents revised as appropriate.

2. Your ethics statement should only appear in the Methods section of your manuscript. If your ethics statement is written in any section besides the Methods, please delete it from any other section.

The ethics statement is present only in the method section.

3. Please note that funding information should not appear in any section or other areas of your manuscript. We will only publish funding information present in the Funding Statement section of the online submission form. Please remove any funding-related text from the manuscript.

Thank you. This has been removed

4. Please upload a copy of Supporting Information Table 1 which you refer to in your text on page 10.

Thank you. We have uploaded copy of all the supporting information, including the tables.

5. If the reviewer comments include a recommendation to cite specific previously published works, please review and evaluate these publications to determine whether they are relevant and should be cited. There is no requirement to cite these works unless the editor has indicated otherwise.

The reviewer comments do not include recommendation to cite specific previously published works.

6. Please review your reference list to ensure that it is complete and correct. If you have cited papers that have been retracted, please include the rationale for doing so in the manuscript text, or remove these references and replace them with relevant current references. Any changes to the reference list should be mentioned in the rebuttal letter that accompanies your revised manuscript. If you need to cite a retracted article, indicate the article’s retracted status in the References list and also include a citation and full reference for the retraction notice.

The reference list is complete and correct. We have not cited papers that have been retracted. We have changed the reference list and added the references 26-36 in response to the review comments on the choice of Osx1-Cre mouse in our studies.

Response to the reviewer:

Reviewer #1: In this interesting study, the authors examined the effects of targeted deletion of Scarb1 in osteoblasts and myeloid cells on bone mass in male and female mice. Using Osx-Cre:Scarb1fl/fl and LysM-Cre:Scarb1fl/fl conditional knockout (cKO), they report no significant differences in trabecular or cortical bone mass in the femur or spine compared to controls. Based on these findings, the authors conclude that Scarb1 in either the osteoblast or myeloid cell lineage is not critical for bone homeostasis.

This study addresses an important and novel question by attempting to identify the specific cell types through which the scavenger receptor Scarb1 mediates the toxic effects of oxidized phospholipids (OxPLs) in bone. The use of male and female mice is valuable because it addresses the possibility of sex dimorphism. However, the study is largely descriptive, relying primarily on DXA and microCT data to demonstrate that such effects are not mediated by Scarb1 expressed in osteoblast progenitors or myeloid cells, without giving any further functional insights into possible underlying mechanisms.

Overall, the manuscript is well-written and presents findings that are relevant to the broader scientific bone research community.

Thank you for the positive comments.

However, several important concerns must be addressed before the manuscript can be considered suitable for publication.

Major

-OxPLs are typically associated with diseased or inflammatory states. Yet, the authors conducted their experiments on relatively young (6-month-old) animals without challenging them with a high-fat or high-cholesterol diet. This represents an important limitation of the study, making it difficult to assess the relevance of the findings to pathological settings. The authors briefly acknowledge this limitation in the Discussion (lines 520-523). The rationale for studying unchallenged animals should be clarified in the Introduction to help readers who are less familiar with the OxPLs field to better understand the study design.

We apologize for the lack of clarity.

The goal of the studies presented in this manuscript was to test if the deleterious effects of PC-OxPLs on bone mass were mediated by their binding to SCARB1 receptor *.* In previous work, we have shown that both male and female transgenic mice expressing a fragment of the antigen-binding domain of the E06 IgM antibody (E06-scFv), which binds and neutralizes PC-OxPLs, exhibited increased cancellous and cortical bone mass at 6 months of age when fed a regular chow diet (Palmieri et al, JBMR 2021, reference #9). These mice showed an increased osteoblast number and bone formation rate at both cancellous and cortical sites, and reduced osteoblast apoptosis in vivo. These results demonstrate that, under physiological conditions, PC-OxPLs inhibit bone formation in young adult mice.

According to our hypothesis, PC-OxPLs could exert their negative effects on bone cells by binding to SCARB1. Therefore, in the studies presented in the present manuscript, we analyzed male and female mice at the same age of the E06-scFv transgenic mice where we observed the increase in bone mass.

We have modified the text (line 92-96 of the introduction).

-In the first experiment (Figure 1), the authors compare calvaria-derived osteoblasts from WT and Scarb1 global KO. This experiment feels somewhat disconnected from the rest of the manuscript, which centers on conditional deletion of Scarb1 in specific bone cells. The rationale behind this experiment is not clearly stated, and the connection to the subsequent cKO studies is underdeveloped. In particular, the authors report significant differences in osteoblast differentiation and proliferation between WT and Scarb1 KO, but do not follow up with similar studies using cells from the Osx1-Cre or LysM-Cre cKO models. This represents a missed opportunity to validate whether these cell-specific deletions replicate the phenotype seen in the global KO.

We apologize for the lack of clarity. In a previous publication (Palmieri et al, JBMR 2021, reference #9) we had shown by gene expression analysis (qPCR) that *Scarb*1 is the most abundant receptor for PC-OxPLs expressed in osteoblast- and osteocyte-like cell lines, as well as in calvaria-derived osteoblasts compared to other PC-OxPLs receptors such as CD36, TLR2, TLR4 and TLR6. In the same publication we had also examined whether *Scarb1* could be involved in the adverse effect of OxLDL on osteoblasts. Silencing *Scarb1* with shRNA protected the calvaria-derived osteoblasts from OxLDL-induced apoptosis. Moreover, bone marrow and calvaria-derived osteoblasts from *Scarb1* KO mice were protected from the pro-apoptotic and anti-differentiating effects of OxLDL. These results strongly suggested that *Scarb1* may be mediating the deleterious effect of OxPL/OxLDL on osteoblastic cells in vitro.

In the present manuscript we expanded these studies and searched in vitro for direct effects of oxidized phospholipids on osteoblast differentiation and proliferation. We again show, with different assays than those used in the previous manuscript (Reference #9) that deletion of *Scarb1* prevents the deleterious effects of oxidized phospholipids in cultured cells.

The new in vitro work presented in this manuscript, along with our published studies, was included as a justification for the *in vivo* work where we conditionally deleted *Scarb1* initially in cells of the osteoblastic lineage and subsequently in cells of the myeloid lineage.

Since conditional deletion of *Scarb1* in either model does not affect the bone phenotype, we decided not to pursue further in vitro work because it will not serve the main purpose of this manuscript which is to understand if the deleterious effects of PC-OxPLs in vivo are mediated by the expression of *Scarb1.*

We have changed the introduction and the first paragraph of the results to make the purpose of this work clearer.

Figure 1A:

1. Osteocalcin and ALP levels appear unusually low for 21-day osteoblast cultures, especially for the WT. The text in Line 220 mentions the use of ΔCt for quantification but refers to a different experiment. The authors should clarify whether ΔCt or ΔΔCt was used in this experiment. If ΔCt was used, data should be re-analyzed using the standard ΔΔCt method, or the decision to use ΔCt should be justified.

We used the ΔCt because it provides a more complete representation of the results. When using ΔΔCt the data are compared to a control normalized to one. This normalization hides the actual values of gene expression. Please note that this method is widely accepted by the scientific community, and we have used it over the last 20 years in numerous publications.

Additionally, there is no detailed description of RNA isolation, cDNA synthesis, or qPCR in the Methods section. A dedicated paragraph should be added.

We thank the reviewer for identifying this omission and we apologize for it. We have added the appropriate paragraph in the method section (Lines 199-212).

Justification for using MRPS2 as a housekeeping gene should also be included, as this is not a commonly used reference gene.

We used MRPS2 as a housekeeping gene because it encodes a mitochondrial ribosomal protein that displays little or no change in expression under a variety of conditions. We have used MRPS2 in several publications (Kim et al, *JCI Insight*, 2020 doi.org/10.1172/jci.insight.138815; Palmieri et al, Aging Cell, 2021, DOI: 10.1111/acel.13442; Campbell et al, Bone, 2024 <https://doi.org/10.1016/j.bone.2024.117181>).

We have added the justification for using MRPS2 as housekeeping gene to the method section (lines 210-212).

2. The authors should have investigated mineralization and ALP activity in the same cell cultures to validate the biological relevance of gene expression data.

ALP activity in both bone marrow-derived osteoblasts and calvaria-derived osteoblasts from WT and *Scarb1* KO mice was published in our previous paper (Palmieri et al, JBMR 2021, reference #9). Herein we used osteocalcin, which is the most specific indicator of osteoblast differentiation.

Figure 1B:

1. A representative figure should be included alongside the quantification to support the proliferation data.

We appreciate the reviewer’s suggestion. However, in our study we used the Cell Proliferation ELISA, BrdU (colorimetric) kit (Roche Diagnostics, Indianapolis, IN, USA), which is a plate-based assay designed to quantify BrdU incorporation by measuring absorbance. In this system, cells are lysed and processed immediately after BrdU labeling to allow antibody binding and colorimetric detection, which precludes the possibility of capturing representative images of proliferating cells. Thus, this assay provides reliable quantitative data but does not generate microscopic images as in immunofluorescence-based BrdU staining.

Minor

-Bone mass is dependent on a balance between bone formation and bone resorption. Even though the overall bone mass is unchanged in both cKO mouse lines, processes of bone formation and resorption could still be altered. However, the authors do not present any data assessing osteoblast or osteoclast differentiation or function. Functional analyses such as bone histomorphometry or in vitro assays using calvaria-derived osteoblasts and bone marrow-derived osteoclasts would strengthen the manuscript and provide a deeper understanding of the cellular consequences of specific Scarb1 deletion.

The main goal of this work was to understand if expression of *Scarb1*  in osteoblasts or in myeloid cells could mediate the negative effects of PC-OxPLs on bone homeostasis at 6 months of age. E06-scFv transgenic mice which, as mentioned above, express the antigen recognition portion of the E06 IgM antibody, that binds and neutralizes PC-OxPLs, exhibited increased cancellous and cortical bone mass at 6 months of age when fed a regular chow diet. These results suggested that, under physiological conditions, PC-OxPLs affect bone homeostasis. If *Scarb1* osteoblasts or myeloid cells mediates the effects of PC-OxPLs under physiological conditions, then bone mass should have been increased in the conditional knockout mice at 6 months of age. The absence of a bone phenotype in the models presented herein indicates that *Scarb1* in these cell types is not involved in the negative effects exerted by PC-OxPLs under physiological conditions.

-Osx1-Cre is known to be aspecific, targeting not only osteoblasts, osteocytes, and hypertrophic chondrocytes, but also bone marrow stromal cells, adipocytes, and perivascular cells. Notably, this Cre line also affects cells outside the skeletal system, including olfactory glomerular cells and subsets of gastric and intestinal epithelium (Chen et al., PLoS One 2014). Given this broad expression pattern, it raises the question: why was Osx1-Cre selected instead of more specific Cre drivers such as Runx2-Cre or Col1a1-Cre, which more selectively target osteoblast-lineage cells

Moreover, Osx1-Cre alone appears to induce a phenotype, as Osx1-Cre controls differ significantly from other control groups in most experiments (Figure 2, Supplementary Figure 3, Supplementary Figure 4, Supplementary Figure 5). While it is understandable that establishing and characterizing another Cre line would be resource- and time-consuming, the authors should explicitly acknowledge the limitations of the Osx1-Cre driver and provide a clearer rationale for its use in both the Methods and Discussion sections.

Osx1-Cre is a well characterized and extensively used Cre driver strain in skeletal research. We selected it because it targets the full range of cells within the osteoblast lineage. Indeed, we have used this Cre driver strain in many earlier publications from our group. (Xiong et al, Nat Med. 2011 doi:10.1038/nm.2448; Almeida et al, J Clin Invest. 2013 doi:10.1172/JCI65910; Jilka et al, J Bone Miner Res. 2014 doi:10.1002/jbmr.2007; Iyer et al, J Clin Invest 2013 doi: 10.1172/JCI68049; Iyer et al, J Biol Chem 2014. doi:[10.1074/jbc.M114.561803](https://doi.org/10.1074/jbc.M114.561803); Ucer et al, J Bone Miner Res. 2015 doi:10.1002/jbmr.2485; Piemontese et al, Scientific Report, 2016 DOI: 10.1038/srep24262; Kim et al, Aging Cell 2017 Doi: 10.1111/acel.12597Aging Cell; Xiong et al, Bone 2018 doi:10.1016/j.bone.2018.04.001; Nookaew et al, JBC <https://doi.org/10.1016/j.jbc.2024.107158>; Ali et al, JBC <https://doi.org/10.1016/j.jbc.2025.110551>). The use of a Cre driver targeting osteoblasts at a later stage of differentiation would have been a logical next step, but only if deletion of Scarb1 with Osx1-Cre had caused a bone phenotype.

Please see lines 119-120 of the revised manuscript.

-The use of three separate control groups (WT, Osx1-Cre, and Scarb1fl/fl), while useful to dissect the effects of the Cre and the loxP sites alone, is uncommon and creates some conflicts in the interpretation of the analyses. In some of the μCT results, for example in Supplementary Figures 3D-F and 4D-F, WT and Scarb1fl/fl controls differ significantly from the cKO, but the authors logically dismiss these differences based on a lack of significance relative to the Osx1-Cre control. If Osx1-Cre introduces changes independent of Scarb1 deletion, then it may be more appropriate to use Osx1-Cre as the sole control. As a suggestion, the comparisons to Osx1-Cre could be shown in the main paper, and the current figures might be moved to the Supplementary. Alternatively, the authors should clearly articulate the reasons for including each control.

Similarly, three control groups were used for the LysM-Cre experiment. Differently than Osx1-Cre, LysM-Cre does not result in a skeletal phenotype alone (Dallas et al., Curr Osteoporos Rep. 2018). As discussed above, a justification for the use of multiple control groups should be provided, or the controls should be streamlined to those that are biologically and statistically appropriate.

Please note that when performing a new conditional knock out experiment, it is essential and an absolute requirement to provide controls for each component of the system, namely the Cre driver alone, the floxed floxed allele alone, and the WT. We have included all three of these controls in our comparison with the conditional knock out mice in both models. Inclusion of this comprehensive set of controls is state-of-the-art for conditional knockout studies.

-In the Abstract, the authors mention that skeletal analysis of Scarb1 KO mice produced contradictory results (lines 33-35). Since the current data indicate that neither the osteoblast nor the myeloid/osteoclast lineage mediates Scarb1 role in bone homeostasis, it would be helpful if the authors could comment on this in the Discussion. Do they have any ideas about what might explain the discrepancies in the earlier findings, or how their results fit into that context?

The preponderance of existing evidence suggests that germline deletion of Scarb1 in mice results in high bone mass. Our results suggest that the effect of Scarb1 deletion on bone homeostasis is unlikely to be related to expression of this receptor in osteoblasts or osteoclasts alone. It is possible that the effects of Scarb1 are complex and mediated by multiple cell types.

We have added the sentence at the end of the Discussion (lines 570-573).

Line 271: sample number reported in Figure 1 caption “C57BL6/J (n=5) and Scarb1 KO mice (n=2)” does not match what is shown in the figure (WT n=3, KO n=3).

Line 274: “4-5-month-old WT and Scarb1 KO mice (n=3/group)”, again numbers do not match with what is shown in the graph.

Please note that for the in vitro studies, we pooled cells from mice of the same genotype and plated the cells in replicates. These are technical replicates, not animal replicates, because we are not looking for changes between animals, but for differences in cells with and without *Scarb1*.

Line 419: “Osx1-Cre mice lost BMD with time […] Figure 4A”. The authors were probably referring to LysM-Cre, not to Osx1-Cre.

Thank you, we have corrected that mistake.

Supplementary Figures 2A-B-C, 3A-B-C, 4A-B-C, 5A-B-C: p values are missing from the Figures and the figure legend.

Thank you. We have added the p values relative to Figs 2 A-C, 3 A-C, 4 A-C in S2 Table and the p values relative to S2 Fig and S7 Fig in S3 Table.

Sincerely,

Elena Ambrogini, M.D., Ph.D.

Associate Professor of Medicine

Director, Division of Endocrinology and Metabolism, Department of Internal Medicine

Associate Professor in the Department of Orthopaedics

University of Arkansas for Medical Sciences

Staff Endocrinologist and Research Scientist

Central Arkansas Veterans Healthcare System
